# An Epistemological View on Risk Exposure Assessment: A Case Study

**DOI:** 10.2174/1874434601711010203

**Published:** 2017-10-31

**Authors:** Daniele Chiffi, Paola Berchialla, Dario Gregori

**Affiliations:** 1 Unit of Biostatistics, Epidemiology and Public Health, Department of Cardiac, Thoracic and Vascular Sciences, University of Padova, Padova, Italy; 2 Department of Clinical and Biological Sciences, University of Torino, Torino, Italy

**Keywords:** Risk assessment, Applied epistemology, Statistical reasoning, Foreign body injuries, Quantitative risk analysis, Exposure, Hazard

## Abstract

**Background:**

Research in Health Risk Assessment is increasingly covering a preeminent role in health care studies. However, risk assessment faces the issue of properly measuring risk exposure.

**Objective:**

The aim of the study has been to tackle some methodological issues regarding the risk assessment analysis in the health field, giving more emphasis to a philosophical and epistemological approach in order to show the difficulties in adopting suitable exposure assessment techniques.

**Method:**

Here, we present a methodological review and a critical discussion of foreign body injuries articles in child population as a case study. A Medline, Econlite and CIS bibliographic search was conducted considering the term “foreign bodies” only in “children” and “risk”. Only English papers are considered. Further research on CDC, CPSC, DGSANCO databases has been performed. Different approaches in risk assessment are reviewed using four case-study papers with the purpose of pointing out their limitations.

**Result:**

Ten papers are retrieved though literature review reporting risk estimate of foreign bodies injuries in children.

**Conclusion:**

Considering that different variables affecting the risk of choking injuries, like intrinsic characteristic of a product or the intensity levels at which children are exposed, and then it seems very difficult to correctly evaluate risk of injuries. For this reason, we have argued for an epistemological and holistic approach toward risk assessment.

## 1 INTRODUCTION

The present paper is aimed at addressing some neglected methodological issues associated to health risk exposure with the perspective of a philosophical and epistemological emphasis. The advantage of using an epistemological perspective lies in the possibility of elucidating problems, undisputedly associated with the determination of risk, which are still and often ignored in the scientific literature. As a case study for our investigation of risk exposure, we will deal with the question of children foreign body (FB) injuries (specifically ingestion/aspiration) which are a public health concern whose solution showed major difficulties in the last 10 years and towards which there is no general consensus among epidemiologists on how to assess their exposure level.

It is well-known that injuries are not accidental events. They are very common and associated to predictable patterns, which can lead in some cases to severe, even fatal, consequences [1]. Many attempts have been made to estimate the risk of FB injuries in children. They range from injury rate estimation, *i.e.* the number of incidents over the children at risk, to more sophisticated quantitative risk assessment approaches that make use of computer simulations with the aim of quantifying the level of exposure to products that can lead to FB injuries. In spite of the fairly wide range of techniques adopted, several epistemological issues are still around, including *(i)* the integration of behavioural variables in risk assessment models, for example how the parents or caregivers’ supervision modifies the exposure; *(ii)* the absence of critical addressing of the completeness of data and its potential impact on risk estimates. Indeed, the assessment of the exposure level to FB injuries is a challenging issue in epidemiology, since many competing methods are used in the epidemiologic literature and especially in case of injuries involving child population. The assessment of the exposure to FB injuries in children is determined by many variables and it is one of the many causes of hospitalization for children.

Section 2 deals with the main ontological and epistemological features of risk. We analytically point out some problem associated to an epistemological investigation on risk. Section 3 presents the logical structure of a scientific theory according to the classical ‘received view’ in the philosophy of science. Section 4 shows an application of this epistemological framework to risk theory, specifically to provide possible insights towards the elusive problem of determining risk exposure for FB injuries in children. Some concluding remarks concerning the validity, limits and conditions of applicability of risk theory to a specific health risk are discussed.

### 1.1 The Nature of Risk and Risk Exposure

From a technical standpoint, risk is often defined as the probability of an outcome in combination with its magnitude and consequences in a specific lapse of time [2]. At any rate, there is not a universally accepted definition of risk and even when defined there might be problems with its acknowledgement. Risk identification is determined by the product *exposure* x *hazard*, which is the cause of a potential *harm*; hence, the acknowledgment and the assessment of the exposure is a key ingredient in risk analysis [3]. Notice that such technical definition can accommodate both desirable and undesirable outcomes, while the intuitive notion of risk is about unwanted (or negative) events. Nonetheless, apart from the technical definition of risk, the intuitive understanding of such notion is influenced by many factors: *the nature of risk* (new risk, number of people exposed to a risk factor, ecological impact with the nature); *the target of risk*, *i.e.* if the risk concerns a general population, then it is conceived as less severe than personal risk; the credibility of *risk communicator* and, finally, *the framing of risk information,* that has to take into account the intuitive ways of thinking of both experts and lay people, which are based on cognitive and emotive heuristics [4]. Notably, risks associated to children’s behaviours are conceived as very threatening. “Emotional arousal reactions also typically occur when the risk is believed to affect children in some special way” [5]. This fact may also explain the difficulties of risk assessment associated to children injuries, since it is not quite clear in the scientific literature how to affectively evaluate FB risk exposure.

From a more general perspective, the debate on the nature of risk ranges from positions where risk has been considered as the same as risk perception, *viz.* risk conceived from a mere subjective point of view [6] to those that assume the risk a state of the world, independent of any subjective dimensions [7].

A useful and classical conceptual map related to the existence of ‘objects’, that we will apply in order to investigate the ontological nature of risk, is the following. The philosophers Frege and Popper distinguished three different levels associated to the ontology of objects [8]. Frege calls them “*realms*”, while Popper calls them “*worlds*”. *World 1* is the realm of concrete physical objects which are objective and their existence is independent of any subject. Mental states belong to *World 2* and they are subjective since a single subject cannot experience the same mental states of another person. Finally, the objects of *World 3* are objective like the objects of *World 1* but they are abstract like the objects of *World 2* and are partially autonomous, *e.g.* once a theorem is proven by a mathematician X then it holds independently of X. Note that “worlds” are not isolated entities, since a world can produce effects on another world, *e.g.* an event of *World 3* such as an injury may cause both a biological damage in *World 1* and psychological effects in *World 2*. From an ontological point of view, saying that the risk is objective means that risk is a concrete object like tables, chairs, *etc*., and this is for sure undesirable or that risk belongs to *World 3*, namely to the realm of objective theories. Of course, this fact does not rule out the possibility of a subjective assessment of risk in *World 2* even if the ontological nature of risk seems to be mainly placed in *World 3*. Risk, like theories and non-empirical entities, is both objective and abstract and, according to realists, its existence precedes any subjective assessment. Such distinction in ontological worlds is usually discharged by *constructivists* who claim that risk is not an objective notion but a concept constructed by a subject or by a society and it cannot be quantifiable. Constructivism concerning risk has the purpose to underline subjective and value-based facets of risk, even if it may face some problems for introducing a sound criterion for comparing different risks and for evaluating risk severity. If risk was merely subjective, then no simple comparison between individuals should seem to be possible, while if it is merely objective than the individual appraisal of the values associated to the risk, specifically to the notion of *harm*, may be lost. Subjective values and epistemic contexts seem to be deeply associated to the notion of *harm* subsequent to a risk. Thus, when evaluating risks there is the interplay between its subjective and objective facets.

Following Kunreuther’s and Slovic’s [9, 10] ideas, we do not consider risk as “real” or “objective” in a mere ontological sense, while we want to hold a view for which the concept of risk is mainly epistemological, namely it deals with the way we come to know in uncertain situations. This view is also classical in philosophy of science. Hempel, in fact, has acknowledged the epistemic nature regarding risk, specifically what he calls ‘inductive risk’, namely the risk of committing a false positive or a false negative error in decision making [8]. The choice of the balance between the two levels of statistical errors is based on some epistemic decisions that Hempel calls “epistemic values”. Accordingly, in place of speaking of realism and constructivism concerning the ontological dimension of risk, it might be preferable to handle the notion of risk within the distinction between *epistemic objectivity* and *epistemic subjectivity*. From an ontological perspective, risk is an object which mainly inhabits *World 3* even if the subjective facets of risk belonging to *World 2* cannot be easily ruled out and the notion of *harm*, which is often associated to risk, belongs to *World 1*, while the epistemological justification of risk might be much more disputed.

In decision theory, uncertainty deals with the lack of knowledge, namely the probabilities of an event are ignored or known with scarce precision, while decisions *under risk* are made when the probability of an event are known [11]. Risk is viewed in decision making as a sort of *certainty of uncertainty*. This is a technical and methodological distinction. Unfortunately, it is often the case that the probabilities of events in decision making are not known with certainty. Hence, we can talk of decision under risk just as a mere level of abstraction without considering our confidence in the probabilistic assessment of the risk. From this it is elicited that either “decisions under risk” and “decisions under uncertainty” fall within an epistemological framework. *Epistemic objectivity* concerning risk does not support the idea that risk presents a special ontological dimension with respect to the other constituents of the world, but it claims that risk is essentially connected with knowledge under uncertainty and the level of uncertainty does not merely depend on its individual assessment. By this perspective, it follows that an agent can be in a risky conditions, even if he/she does not know it, namely the existence of risk does not require any mental construction of uncertainty, contrary to what held, for instance, by [12] Aven and Renn [12]. It is remarkable that the objectivist perspective on risk is essentially probabilistic, while the holders of an *epistemic subjectivism on risk* claim that no single attribute is a necessary condition for the existence of risk, since risk equates to individual ‘risk perception’. But this fact implies a collapse between elements of *World 2* with elements of *World 3*, while we have pointed out that there is interplay between worlds concerning some facets of risk. Instead, other subjectivists claim that risk cannot be justified objectively but merely in a particular context. Contextualists about risk seem to maintain that there are risks in which there is no place for probabilities [13] even if, from a more broad philosophical point of view, it is possible to be a contextualist without denying the use of probabilities and quantitative measures.

In any case, the objective dimension of risk is mainly placed in *World 3*, the realm of objective scientific theories, and that is why we will try to apply the classical structure of scientific theories to risk theory.

### 1.2 Structure of a Theory and Epistemological Holism

In conformity with the ‘received view in philosophy of science’ a theory is composed by a mathematical apparatus of logical and specific axioms, auxiliary hypotheses, definitions, *etc*. and “corresponding rules” which partially interpret theoretical elements with observative ones in a model. The choice of the model is partially a subjective issue, but many objective demands need to be fulfilled in order to construct a model which can handle data at an optimal level of material adequacy and in accordance with some methodological constraints. The individuation of the model and the choice of the auxiliary hypotheses within a theory can lead towards very different results. The aim of a theory is not merely to describe the world, but to predict empirical observations, namely every theory mainly has a normative and predictive function. From a logical point of view, from a set of premises (*e.g.* laws, hypotheses and initial conditions) one can ‘atomistically’ derive some consequences corresponding to observative statements which must preserve truth in the logical steps by means of sound inferences. By contrast, if the consequences of a set of premises of a theory turn out to be false, then the falsification of the premises is merely of holistic type, *i.e.* one cannot decide which condition(s) expressed in the premises is/are false. By way of example, let’s consider a very simple theory with a law A, an auxiliary hypothesis H and the initial condition C. Let us assume that the conjunction of A, H and C entails an observative statement O. If O turns out to be false and assuming that C has been empirically verified, then it is impossible to determine which one between A or H (or both) is false, since from not-O it is only possible to derive not-(A & H), that is equivalent with (not-A or not-H). This fact is named “epistemological holism” or “Duhem thesis” and it is a fundamental feature concerning the falsificability of scientific theories [14] which has been already used in applied epistemology in a case of forensic statistics [15]. When dealing with probabilistic hypotheses it is possible to compute and evaluate the impact of two different hypotheses H_1_ and H_2_ within a theory by means of Bayes Theorem. If the difference in the probability of H_1_ with respect to the probability of H_1_| not-O is greater than the difference in the probability of H_2_ with respect to the probability of H_2_| not-O, then not-O falsifies H_2_ more than H_1_. Thus, the necessity to compare the impact of different hypotheses in a model of a theory is a key ingredient for an accurate epistemological analysis of the theories. In order to evaluate the felicity of the application of these classical methodological remarks in a case of health risk assessment, we will explore the epistemological limitations of some studies relying on different models for evaluating the elusive issue of risk exposure to FB injuries in children. We underline the importance of the selection of hypotheses in order to provide a theoretical framework to be applied to assess risk exposure.

### 1.3 Limitations of Statistical Reasoning in Risk Exposure: FB-Injury Risk Assessment as a Case Study

The inhalation of FBs into the upper airways can be a serious event, occasionally resulting in lethal outcomes, and frequently having considerable social, psychological and economic consequences. Differently from other injuries, the seriousness of FB injuries is often disputed since many of them are self-resolving. As a way of example, if a gap between the exposure and the selection exists, then it is possible to incur something similar to a prevalence-incidence bias (also named as ‘Neyman bias’). This bias is determined by the inclusion of prevalent cases in the clinical studies. Sackett observed that this bias is associated to “a late look at those exposed (or affected) early will miss fatal and other short episodes, plus mild or silent cases and cases in which evidence of exposure disappears with disease onset” [16] Namely, an association connected to the exposure of a risk factor may be spurious, *e.g.*, a hypothetical association inferred by hospital records between the exposure to FBs and mortality may be falsified since the possibility that some children may have experienced injuries of minor severity not requiring hospitalization. As a consequence, the level of association between a risk factor and an outcome can be easily underrated. Indeed, disease duration and severity can modify the assessment of the association between determinants and outcome when exposure is not adequately analysed and thus possibly determining notable differences on interpreting risk exposure among studies.

Indeed, the assessment of the incidence and the risk associated to FB injuries is methodologically (and legally) challenging. Notably, the issue of the estimation of risk of Food Products Containing Inedibles (FPCIs) for children has been discussed during the regulatory debate in Europe around the revision of the “Toy”-Directive (88/378/EE). Without entering the specific merit of the issue, which has been reported in deep elsewhere [17] our concern here is targeted toward the appropriateness and logical sustainability of the risk estimates as provided supporting one or the other position on that debate, showing that all of them were far from being a sound application of the epistemological principles of risk evaluation as highlighted in the previous sections.

A critical review of methodologies encountered in the literature is thus presented and critically discussed from an epistemological perspective. The objective of this paper is showing that estimates reported have been fraught with methodological weakness associated to probabilistic reasoning, often based on fallacious risk assessment, by means of an application of epistemological tools to the methodology of risk exposure to FB injuries.

## 2 METHODS

In order to evaluate exposure in FB-injuries studies in children population, a Medline, Econlite and CIS search was conducted using the term “foreign bodies” limited to “children” and “risk”. Only English written papers were taken into consideration. Additional search on CDC, CPSC, DGSANCO databases was performed.

## 3 RESULTS

Our bibliographical research retrieves a total of ten papers that reported a risk estimate of the injuries due to foreign bodies in children [18-27]. In the following, the different approaches to the problem of risk assessment are methodologically reviewed using four case-study papers with the purpose of pointing out their limitations.

## 4 DISCUSSION

### 4.1 Quantitative Risk Analysis

Rider *et al.* [25] analysed the risk of choking associated with any given consumer product using the Quantitative Risk Analysis (QRA) approach [25] Monte Carlo simulation methods were used to generate estimates of product-related risk and an example of the methodology is provided for the FPCI.

The QRA is based on the equation risk which is given by the product of Hazard and Exposure. In the proposed approach, the probability of an injury is determined by the likelihood that a series of critical steps (“critical paths to injury”) occur. For example, considering the case of a choking death due to asphyxiation, the critical path to injury includes the following events: that (*i*) the child has access to the product, that (*ii*) at least a part of the product reaches the rear of the oral cavity or enters the throat, and (*iii*) that the object obstructs the airway for a length of time adequate for a significant portion of the brain cells to die. The probability for each step in the critical path was based upon statistical analysis of trends in historical and empirical data. Furthermore using Human Factors Analysis [28] the impact of size and kinetic behaviours of children along with their ability to access hazardous product characteristics were quantified. Human Factors Analysis is the study of the physical interaction between a consumer and a product. Consumer-product interactions can result in a variety of injuries, including suffocation, burn injuries and small parts injuries, *i.e.* injuries due to small parts for example by accidental spikes and sharp edges.

Different age groups of children have different quantifiable probabilities of being involved in an incident, injury or fatality associated with a given product. Making use of a “foreseeable analysis”, in order to evaluate the potential effects of an activity in a specific context, critical activities such as mouthing, which could result in an injury, can be quantified as a function of the children age and also of specific product characteristics.

A first inconsistency in the analysis that Rider *et al.* presented is about the probability of mouthing they estimated. First of all, such model needs the probability of purchasing and delivering a FPCI to a child in the US (since FPCI are banned from US). Instead, in the QRA results mouthing incidents were estimated based on the mouthing incident probability which is, for a 3-year-old child, approximately 0.002. This value means that a 3-year-old child, over an entire year of life, during which he presumably had many occasions to come into contact with pieces of paper, small stones, pieces of plastic, sand, *etc*. is exposed to a risk of two out of every thousand to put something into his mouth. This probability, which is a high probability, is attributed to the contact of a child with only one FPCI, overestimating than the risk of mouthing. The same reasoning applies for the risk of choking.

### 4.2 Injuries from Food Products Containing Inedibles

Morra and Passali [29] pointed out the weakness in the estimation of risk of injury due to FPCI in a report produced by Petridou *et al*. [23]. On the basis of three minor ingestion accidents involving FPCIs, Petridou concluded that “a total of 2000 FPCI injuries are expected to happen each year in the European Union” [24]. A major concern in the estimation is about data. Data were collected only in the area of Great Athens during a 4-month period. It is well known that Greece has by far the highest death rate from choking of children under 10 in the European Community; this may represent atypical behaviour. For these reasons, the estimate of 2000 injuries each year is probably too high. Otherwise the computation of the 95% confidence interval gives a very large interval, equal to (0–5918), meaning that it would not be completely unlikely that no injuries will be reported next year, but it is also not completely impossible that we would have almost 6000 accidents. This simple consideration shows that the precision of the estimate reported in the paper is very poor as pointed out by Croux^1^.

### 4.3 Inedibles in Food Product Packaging – Draft Final Report prepared for STOA, European Parliament

In a detailed report developed by RPA (Risk & Policy Analysts Limited) [26], it has been undertook a consultation exercise to provide details on the nature and the number of FPCI (Food Products Containing Inedibles) incidents. They used the risk equation (hazard) x (exposure). Exposure was assessed by estimating population at risk and the FPCI marketed within EU The latter parameter is estimated in the order of five billion per year. Although, RPA emphasized it was a very uncertain figure based on extrapolation of limited data gained. To estimate the number of FPCI injuries, the assumption that all children are exposed to the same degree of hazard, irrespective to their country of residence was made. Furthermore, the analysis was based on the hypothesis that 5% of all choking incidents were caused by toys and 1% of all choking incidents caused by toys involved FPCIs.

The overall non-fatal choking incidents resulting in admission to hospital was of the order of 100 per 100,000 per year. But using this figure to estimate the number of FPCI incidents resulted in a discrepancy among predicted and reported injuries. In particular, France, Italy and UK had fewer reported incidents than predicted while Germany and Greece actually had more than predicted. The reason probably relies in the fact that levels of exposure strongly depend on country.

(i) *Development of a Method Allowing to Define Security Rules for Particular Classes of* *Products, to be Enforced through Technical Standards by European Bodies under Mandate of the European Commission* – Final Report

In this final report [20] (Deheuvels 2003) it has been proposed product-related risk assessment based upon accidental data if and only if the following statistical counts are available:

- the annual number of accidents observed in relation with the product;
- the number of individuals composing the population exposed to the product;
- the number of product items set annually in use.

The main limitation is the “if” condition. Exposure is given by parameters (b) and (c). But the number of individuals composing the population exposed to the product is a roughly approximation, which does not take into account levels of accessibility to a product as function of the children age and product features (for example the mouthing activity of children under three years old). Namely, (a), (b) and (c) are jointly necessary but not sufficient conditions for the assessment of risk.

The above examples of studies concerning FB injuries in children are related to the question of the structure of a theory and to epistemological holism. Namely, they show that i) auxiliary hypotheses should be made explicit in the model (*e.g.* the differences in incidences of FB injuries among different countries) in order to evaluate their probabilistic degree of potential falsification and subsequently ii) only when auxiliary hypotheses closely mirror reality, then the predictive consequences of the model may be sound.

## CONCLUSION

We have pointed out the indispensability of the epistemic facets of risk. On this basis, we have argued for an epistemological approach toward risk and risk exposure rather than an ontological one. We have focused as a case study on the interpretation of FB risk exposure in children, since there is no general consensus among epidemiologists on how to determine risk assessment associated with this health-related phenomenon. For this reason, we have critically analysed the literature on a specific type of FB injuries in children from an epistemological perspective. It has been remarked that in the aforementioned articles on FB injuries there is no awareness that the choice of the model and the justification of the auxiliary hypotheses regarding the determination of the risk exposure, *e.g.* the level of accessibility of a product to a child may lead towards controversial assessments in risk evaluation. Moreover, the choice of the relevant homogeneous subpopulations and plausible hypotheses is necessary for soundly assess the exposure, which may vary considerably in different countries and coherently with some specific hypotheses accepted in a model. This partial lack of methodological tools is the main cause of the not correct estimations of the injury rate in the articles taken into consideration. We observed, on the contrary, that exposure assessment in evaluating FB risk requires many methodological constrains which can affect the interpretation of epidemiological data concerning FB injuries.

We have emphasised that it is not easy to establish precisely the risk inherent to FB injuries in children because of the fallacies and biases frequently associated with the determination of risk exposure. The major issue is the number of the variables affecting the risk. Many of them are intrinsic characteristic of a given product, such as its shape or its consistency, while others are related to the intensity levels at which children are exposed and may be difficult to assess. Indeed, it is necessary to know the number of the marketed products, the frequency of their use and the intensity of hazardous behaviours (for example the intensity of mouthing) and formulate methodologically plausible hypotheses in order to assess the levels of risk for FB injuries in children.

On the other hand, data about FB injuries in children come from hospital discharge records and a basic question is thus the following: how many real cases of injuries occurred? On the basis of official records, the number of injuries is underreported since the accidents of minor severity are indeed self-resolved. Being all these accidents lost at observation, the overall risk is grossly underestimated.

Moreover, we have pointed out that many implicit epistemological assumptions lay behind the choice of the probabilistic model and auxiliary hypotheses that must be taken into greater account when evaluating risk exposure as well as the possibility to incur in a situation of epistemological holism^2^.

In conclusion, an epistemological analysis on the structure of a theory in accordance with the ‘received view’ in philosophy of science has guided our investigation in order to provide an interpretation of the fallacies associated to the assessment of risk exposures for a specific health risk. In this way, we have provided an example in which applied epistemology shows its significance and scopes when dealing with a specific but elusive epidemiologic case.

## Footnotes

1 Croux, C. (1998): Comments on the Paper “Injuries from Food Products Containing Inedibles –”, Institut de Statistique, Université Libre de Bruxelles, Belgium.

2 The same epistemological considerations may be applied, for instance, to the evaluation of the effectiveness of risk communication. Risk communication is critically dependent on the confidence placed in the risk assessment and divergent epistemological stances can also implies the lack of confidence engendered by the use of quantitative risk assessments. To communicate effectively with a general audience outside the risk assessment community, it is important risk communicators recognize and take into account the epistemological issues that affect the way their communications will be interpreted.

## LIST OF ABBREVIATIONS

FB: = (Foreign body injuries)
QRA: = (Quantitative Risk Analysis)
FPCI: = (Food Products Containing Inedibles)
